# Academic achievement of twins and singletons in early adulthood: Taiwanese cohort study

**DOI:** 10.1136/bmj.a438

**Published:** 2008-07-31

**Authors:** Meng-Ting Tsou, Meng-Wen Tsou, Ming-Ping Wu, Jin-Tan Liu

**Affiliations:** 1 Department of Family Medicine, Mackay Memorial Hospital, Taiwan; 2 Nursing and Management College, Mackay Medicine, Taiwan; 3 Department of International Trade, Tamkang University, Taiwan; 4 Division of Urogynecology and Pelvic Floor Reconstruction, Department of Obstetrics and Gynecology, Chi Mei Foundation Hospital, Taiwan; 5 Department of Economics, National Taiwan University, Taiwan

## Abstract

**Objectives** To examine the long term effects of low birth weight on academic achievements in twins and singletons and to determine whether the academic achievement of twins in early adulthood is inferior to that of singletons.

**Design** Cohort study.

**Setting** Taiwanese nationwide register of academic outcome.

**Participants** A cohort of 218 972 singletons and 1687 twins born in Taiwan, 1983-5.

**Main outcome measure** College attendance and test scores in the college joint entrance examinations.

**Results** After adjustment for birth weight, gestational age, birth order, and sex and the sociodemographic characteristics of the parents, twins were found to have significantly lower mean test scores than singletons in Chinese, mathematics, and natural science, as well as a 2.2% lower probability of attending college. Low birthweight twins had an 8.5% lower probability of college attendance than normal weight twins, while low birthweight singletons had only a 3.2% lower probability. The negative effects of low birth weight on the test scores in English and mathematics were substantially greater for twins than for singletons. The twin pair analysis showed that the association between birth weight and academic achievement scores, which existed for opposite sex twin pairs, was not discernible for same sex twin pairs, indicating that birth weight might partly reflect other underlying genetic variations.

**Conclusions** These data support the proposition that twins perform less well academically than singletons. Low birth weight has a negative association with subsequent academic achievement in early adulthood, with the effect being stronger for twins than for singletons. The association between birth weight and academic performance might be partly attributable to genetic factors.

## Introduction

The cognitive disadvantage for twins during childhood has been well documented. Most of the earlier studies, which used cohorts from the 1950s and 1960s, found that cognitive ability (as measured by verbal reasoning scores and test scores in word knowledge, reading, and numeracy) was lower among twins than singletons.^1^ ^2^ ^3^ ^4^ Although cohort analyses of the entire samples in these studies did provide relatively consistent evidence, conflicting results were nevertheless reported in family based studies.

A study carried out in the Netherlands with 260 adult twins and their 98 singleton siblings reported little difference in intelligence between the samples.^5^ In contrast, a large sample study of primary students born in Scotland in 1950-6 found that, at the ages of 7 and 9, twins had substantially lower IQ scores than their singleton siblings, even after adjustment for potential confounding factors within the families.^6^ A more recent study, which used nationwide registers of ninth grade (aged 15-16) children born in Denmark in 1986-8, concluded that the academic performance of twins and singletons was quite similar.^7^

Cognitive functioning and intellectual performance are developed through a mixture of genetic and environmental effects.^8^ ^9^ The tendency towards lower intelligence among twins might be attributable to both the suboptimal intrauterine environment and the subsequent family environment.^5^ ^6^ ^10^ A shared fetal environment might lead to impairment in brain growth, thereby giving rise to adverse effects on cognitive development. Furthermore, compared with singletons, twins might experience greater limitations on resources, as well as heightened competition, within the family environment in which they are brought up. The influence of genetic factors in determining childhood IQ levels has also been examined by using data on pairs of twins, with a study of 170 same sex twin pairs reporting that the association between birth weight and IQ could be attributable to underlying genetic factors.^11^

We used the Taiwanese nationwide registers of academic outcomes, which is based on the results of the college joint entrance examination, to compare performance levels between twins and singletons. We undertook separate analyses of the effects of low birth weight on long term academic outcomes for twins and singletons and the effects on twin pairs.

## Methods

### Data

We linked the Taiwanese nationwide academic outcomes in the college joint entrance examinations for 2002-3 to national birth certificate record data for 1983-5. We used the outcomes of the examination to measure academic achievement in early adulthood because we did not have data on IQ scores. The examination, which is generally considered to be the main channel for the entry of Taiwanese students into universities and colleges, also represents a pivotal point in determining an individual’s future educational attainment. National universities in Taiwan are regarded as being more prestigious than private universities, although the tuition fees are lower.

In accordance with the education system in Taiwan, the college joint examinations are taken at 18. Children born between September 1983 and August 1984 should have taken the entrance examinations held in 2002, while those born between September 1984 and August 1985 should have done so in 2003.

Comprehensive details on sex, gestational age, birth weight, birth order, birth place, and multiple birth status are contained within the birth certificate files, along with details on the age, years of education, and working status of the parents at the time of the birth. The college joint entrance examination files contain information on whether the student was enrolled in a college and the test scores obtained (on a scale of 0-15) in the five major subjects (Chinese, English, mathematics, natural science, and social science). The overall test score was measured as the sum of the five subjects.

We used the unique personal identifier to merge the college joint entrance examination files with the birth certificate files and restricted our sample to those born from September 1983 to August 1985. After excluding those with missing data on parental characteristics, we were left with 220 659 observations for our analysis, comprising 1687 twins and 218 972 singletons.

### Statistical methods

We used the univariate comparison method to compare academic performance of twins and singletons. To account for potential confounding factors, we then used logistic regression analysis to determine the probability of college attendance (dichotomous outcome) and multiple linear regression analysis for the test scores (continuous outcomes), with adjustment for sex, gestational age, birth weight, and birth order and socioeconomic characteristics of the parents. We examined the impact of low birth weight on long term academic outcomes by estimating the regressions separately for twins and singletons. Finally, focusing on the twin pair sample, we used the twin fixed effect approach to examine the effect of birth weight on academic achievement to provide further control of unobserved parental and environmental factors across families.

Potential confounders in the comparison of academic outcomes between twins and singletons included low birth weight, gestational age, birth order, and sex and the parents’ socioeconomic status. Low birth weight is a recognised and well documented predictor of childhood cognition and educational attainment.^6^ ^12^ ^13^ ^14^ Another important confounder is gestational age, which enables us to distinguish between low birth weight caused by preterm delivery and that resulting from intrauterine growth restriction.^13^

While the preferences of the parents between sons or daughters might affect parental investment in the children,^14^ children born later were generally found to have poorer educational outcomes than those born earlier.^14^ ^15^ The socioeconomic environment in which a child is reared will also affect the child’s developmental and educational attainment—for example, highly educated parents might invest more in the development and education of their children than parents with lower levels of education, while parental education itself can also reflect effects of unobserved genetic factors on the academic performance of their children. Families in which parents are better educated or employed within the public sector, or both, might also be less financially constrained than those with lower education levels or those employed within the private sector.^14^ ^15^

## Results

Table 1 shows the summary statistics on the characteristics of the twins and the singletons[Table tbl1]. The average birth weight of twins (2570 g) was substantially lower than that of the singletons (3287 g). Over 40% of the twin births were classified as low birth weight (<2500 g) compared with only 3% of singletons. The gestational period tended to be shorter for twins (38.1 weeks) than singletons (39.7 weeks), while birth order was higher for twins (1.9) than singletons (1.8). On average, parents of twins were older at the time of birth than parents of singletons (mothers 27.6 *v* 26.8 years; fathers 30.7 *v* 29.8 years) and had more years of education (mothers 10.9 *v* 10.4 years; fathers 11.9 *v* 11.4 years).

**Table 1 tbl1:** Characteristics of twins and singletons in Taiwan. Figures are means (SD) unless stated otherwise

Variables	Singletons	Twins
No of live births	218 972	1687
No (%) male	109 619 (50.1)	823 (48.8)
Mean (SD) birth weight (g)	3287 (440)	2570 (473)
No (%) with low birth weight (<2500 g)	6300 (2.9)	691 (41.0)
No (%) premature (≤37 weeks)	9901 (4.5)	541 (32.1)
Gestational age (weeks)	39.7 (1.3)	38.1 (2.2)
Birth order	1.8 (0.9)	1.9 (1.0)
Maternal age (years)	26.8 (3.7)	27.6 (3.9)
Paternal age (years)	29.8 (4.5)	30.7 (4.8)
Maternal education (years)	10.4 (3.3)	10.9 (3.2)
Paternal education (years)	11.4 (3.3)	11.9 (3.3)
No (%) employed in public sector:		
Mothers	19 949 (9.1)	194 (11.5)
Fathers	191 148 (12.7)	237 (14.0)

After adjustment for sex, gestational age, birth weight, and birth order and the age, education, and working status of the parents at the time of the birth, logit estimates indicated that twins had a 2.2% lower probability of college attendance than singletons (table 2).[Table tbl2] Table 3 shows the adjusted mean difference in test scores between twins and singletons. Twins had significantly lower mean scores in Chinese, mathematics, and natural science, although there was little difference in the mean scores for English and social science. The overall test score was significantly lower for twins than for singletons.

**Table 2 tbl2:** Odds for college attendance, crude and adjusted odd ratios (95% CI), and marginal effects

	Odds*	Odds ratio (95%CI)		Marginal effect†
		Crude	Adjusted	
Birth:				
Singleton	2.14	1	1	—
Twin	1.86	0.87 (0.78 to 0.96)	0.89 (0.80 to 0.98)	−0.022
Birth weight:				
Normal	2.15	1	1	—
Low	1.83	0.85 (0.81 to 0.89)	0.85 (0.80 to 0.90)	−0.035
Sex:				
Female	2.18	1	1	—
Male	2.11	0.97 (0.95 to 0.99)	0.94 (0.92 to 0.96)	−0.011
Gestation age (weeks):				
≤37	2.13	1	1	—
38-42	2.14	1.01 (0.96 to 1.05)	0.98 (0.93 to 1.03)	−0.002
≥43	2.40	1.13 (1.01 to 1.26)	1.10 (0.97 to 1.24)	0.013
Birth order:				
1	2.19	1	1	—
2	2.20	1.00 (0.98 to 1.02)	0.96 (0.94 to 0.98)	−0.006
3	0.35	0.91 (0.89 to 0.94)	0.85 (0.83 to 0.88)	−0.022
≥4	0.10	0.80 (0.77 to 0.84)	0.76 (0.72 to 0.80)	−0.041
Maternal age (years):				
18-29	2.07	1	1	—
30-39	2.41	1.16 (1.14 to 1.19)	1.12 (1.09 to 1.15)	0.016
≥40	1.96	0.95 (0.80 to 1.12)	1.16 (0.96 to 1.41)	0.022
Paternal age (years):				
18-29	1.99	1	1	—
30-39	2.34	1.18 (1.16 to 1.20)	1.19 (1.16 to 1.21)	0.025
≥40	2.03	1.02 (0.96 to 1.08)	1.17 (1.09 to 1.24)	0.012
Maternal education (years):				
Elementary	1.79	1	1	—
Junior high	1.80	1.01 (0.98 to 1.04)	0.99 (0.96 to 1.02)	−0.002
Senior high	2.19	1.22 (1.20 to 1.25)	1.13 (1.10 to 1.16)	0.023
College or above	3.23	1.81 (1.75 to 1.86)	1.53 (1.47 to 1.60)	0.078
Paternal education (years):				
Elementary	1.70	1	1	—
Junior high	1.79	1.05 (1.02 to 1.09)	1.06 (1.02 to 1.09)	0.012
Senior high	2.04	1.20 (1.16 to 1.23)	1.13 (1.10 to 1.17)	0.028
College or above	2.88	1.69 (1.64 to 1.74)	1.41 (1.36 to 1.47)	0.070
Mother employed in public sector:				
No	2.05	1	1	—
Yes	3.44	1.67 (1.62 to 1.73)	1.15 (1.11 to 1.20)	0.021
Father employed in public sector:				
No	2.06	1	1	—
Yes	2.90	1.41 (1.37 to 1.45)	1.07 (1.03 to 1.10)	0.034

*Ratio of probability of attending college to probability of not attending college.

†Partial derivative of probability function with respect to each covariate.

**Table 3 tbl3:** Descriptive statistics and adjusted mean differences (95% CI) in test scores between twins and singletons

	Singletons			Twins		Estimated coefficient* (95% CI)
	Mean (SD)	Median (IQR)		Mean (SD)	Median (IQR)	
Chinese	10.9 (2.0)	11 (10-12)		10.8 (2.0)	11 (10-12)	−0.108 (−0.202 to −0.014)
English	9.1 (3.2)	9 (6-12)		9.1 (3.3)	9 (6-12)	0.002 (−0.149 to 0.154)
Mathematics	6.9 (3.4)	7 (4-9)		6.7 (3.4)	6 (4-9)	−0.168 (−0.326 to −0.010)
Natural science	10.0 (2.2)	10 (8-12)		9.8 (2.2)	10 (8-11)	−0.174 (−0.277 to −0.070)
Social science	12.2 (1.7)	12 (11-14)		12.1 (1.8)	12 (11-13)	−0.071 (−0.156 to −0.013)
Overall test score	49.1 (9.7)	49 (42-56)		48.5 (9.8)	49 (41-56)	−0.528 (−0.928 to −0.075)

IQR=interquartile range.

*Adjusted for birth weight, gestation age, birth order, and sex and parents’ age, education, and work status at time of birth.

When we carried out separate regression estimations for twins and singletons, we found that low birth weight was an important predictor of long term academic performance. Low birthweight twins had an 8.5% lower probability of college attendance than their normal birth weight counterparts, while low birthweight singletons had only a 3.2% lower probability of college attendance.

Within the sample of twins, those with low birth weight had significantly lower test scores in English and mathematics than twins with normal birth weight; the estimated coefficients indicating that low birth weight was responsible for a substantial reduction, of 0.48, in the scores for these two subjects. There was little association between low birth weight and the scores for Chinese, social science, and natural science.

Within the sample of singletons, there was a significant and negative association between low birth weight and the test scores for all subjects. The effects of low birth weight on the singletons tended to be smaller than those for the twins.

To identify whether there is any association between birth weight and levels of academic performance within twin pairs we used the twin fixed effect approach^16^ ^17^ to regress the differences within twins in birth weight on the differences within twins in the test scores. This enabled us to account for unobserved heterogeneity across families.

The twin pair analysis included 377 pairs of twins, of which 316 were same sex pairs. The reduced sample size reflects the possibility that twins within the same family might not have taken the college entrance examinations together in the same year—for example, if one of the twins was too handicapped to take the examination. Given that we were unable to distinguish between dizygotic twins and monozygotic twins, by separating the sample into opposite sex twins (which are clearly not monozygotic) and same sex twins (which account for most monozygotic twins) we were able to investigate whether the effect of birth weight might partly reflect genetic differences between the twins.

For the whole sample of twin pairs, twins with higher birth weight performed better in mathematics: with an increase of 100 g in birth weight, there was a corresponding increase of 0.09 in the score for mathematics. Nevertheless, after reclassifying the twin sample into same sex and opposite sex twin pairs, we found that although birth weight in opposite sex pairs did have a significant effect on the scores for English and mathematics, the same effect was not discernible for same sex pairs. 

## Discussion

Twins seem to be at long term educational disadvantage. In this large study in Taiwan, we merged the college joint entrance examinations files and birth record files and found that twins had lower academic achievement than singletons. Even after we controlled for potential confounding factors, the test scores in Chinese, mathematics, and natural science and the likelihood of college attendance were all significantly lower for twins than for singletons. This implies that twins are more likely to have lower educational attainment than singletons, which might ultimately limit their future earnings potential.^18^ ^19^

Our results agree with several previous studies in which the performance of young twins was found to be inferior to that of singletons.^1^ ^2^ ^3^ ^4^ Nevertheless, the results differ from those of a recent Danish study in which it was indicated that both twins and singletons showed similar academic performance during adolescence.^7^ The difference in academic outcomes between twins and singletons might be attributable to the impaired fetal growth of twins and greater limitations on resources for those families within which twins are reared.^5^ ^6^ ^8^ ^9^ ^10^

Our results indicate that the effect of low birth weight on academic performance persists into early adulthood. Low birth weight might be responsible for some impairment in brain development, and could result in lower intellectual performance.^9^ ^20^ ^21^ ^22^ ^23^ Given that the incidence of low birth weight is substantially higher among twins and that the negative effect of low birth weight on academic performance is greater for twins than for singletons, we reaffirm the view that low fetal growth might well result in a long term educational disadvantage for twins.^8^ ^9^

The results of our twin pair analysis are in line with the findings of an earlier study undertaken in the Netherlands on 170 same sex twin pairs; in their examination of differences within pairs of twins in birth weight and IQ at ages 7 and 10, they found a positive association only for dizygotic twin pairs and not for monozygotic twin pairs.^11^ We cannot exclude the possibility that the positive effect of birth weight, which was discernible in our opposite sex twin pairs, might be related to sex effects—for example, boys usually have higher birth weight than girls, and males quite often have higher scores than females in mathematics.

### Strengths and limitations

Our study had several factors that strengthened the importance of our conclusions. Firstly, the large sample size and high quality of our national datasets permit powerful comparisons between the academic performance of twins and singletons. Secondly, with our sample of births from the 1980s, we provide initial evidence from a more recent cohort in an Asian country. Finally, to determine the effect of birth weight on long term academic achievement, we use not only a multivariate regression approach to the whole cohort analysis but also a twin fixed effect approach to our twin pair analysis.

The limitations of our data, however, are the possibility of selection bias arising as those who did not take the college joint entrance examinations would be excluded from our analysis; the absence of information on several potential confounding factors, such as admission to hospital or morbidity of a child during its early neonatal stage, whether the child was born after assisted conception, the quality of care during early childhood, the size of the family, and the peer group effect on academic achievement; and our inability to distinguish dizygotic twins from monozygotic twins in the same sex twin pairs. Also, although 2172 subjects had missing data on parental characteristics, the descriptive statistics of the birth characteristics for twins and singletons among these subjects were generally similar to those for our whole cohort analysis. Thus, we think this is unlikely to cause any serious bias to our basic estimates.

Although the validity of twin pair analysis has been criticised on the grounds of the lack of generalisability, it does provide a powerful means of controlling for fixed parental and familial characteristics, as well as certain genetic factors shared by twins.^16^ ^17^ ^24^ Our comparisons within twins suggest that the association between birth weight and academic performance might be partly explained by genetic factors, which leads us to one important caveat, that the estimates of birth weight based on the entire population might be biased upward as a result of genetic variations.

**What is already known on this topic**

Some early studies reported that twins had lower IQs than singletons during childhood, while more recent evidence indicates that academic performance during adolescence is similar for twins and singletons
Birth weight had a positive association with IQ in older cohorts, whereas little effect has been reported on academic performance in more recent cohorts

**What this study adds**

In Taiwan, twins have lower academic achievement scores than singletons and are less likely to go to college
Low birth weight has a negative effect on long term academic performance for both twins and singletons, though the effect is stronger for twins
Comparisons of effects of birth weight across same sex and opposite sex twins suggest that birth weight might partly reflect other underlying genetic variations

## Notes

Ethical approval: Ministry of Interior Affairs and Ministry of Education in Taiwan.

Provenance and peer review: Not commissioned; externally peer reviewed.

## Notes

**Cite this as:** *BMJ* 2008;337:a438
